# *P. gingivalis* in Periodontal Disease and Atherosclerosis – Scenes of Action for Antimicrobial Peptides and Complement

**DOI:** 10.3389/fimmu.2015.00045

**Published:** 2015-02-10

**Authors:** Mehak Hussain, Cordula M. Stover, Aline Dupont

**Affiliations:** ^1^Department of Infection, Immunity and Inflammation, University of Leicester, Leicester, UK; ^2^Institute for Medical Microbiology and Hospital Epidemiology, Hannover Medical School, Hannover, Germany

**Keywords:** *P. gingivalis*, complement system, antimicrobial peptides, periodontitis, atherosclerosis

## Abstract

According to the NHS, it is estimated that over 50% of the adult population are, to some extent, affected by gum disease and approximately 15% of UK population have been diagnosed with severe periodontitis. Periodontitis, a chronic polymicrobial disease of the gums, causes inflammation in its milder form, whereas in its severe form affects the surrounding tissues and can result in tooth loss. During periodontitis, plaque accumulates and sits between the junctional epithelium and the tooth itself, resulting in inflammation and the formation of a periodontal pocket. An interface is formed directly between the subgingival bacteria and the junctional epithelial cells. Bacterial pathogens commonly associated with periodontal disease are, among others, *Porphyromonas gingivalis*, *Tannerella forsythia*, and *Treponema denticola*, together known as the “red complex.” This review will mostly concentrate on the role of *P. gingivalis*, a Gram-negative anaerobic bacterium and one of the major and most studied contributors of this disease. Because periodontal disease is associated with the development of atherosclerosis, it is important to understand the local immune response to *P. gingivalis*. Innate immune players, in particular, complement and antimicrobial peptides and their effects with regard to *P. gingivalis* during periodontitis and in the development of atherosclerosis will be presented.

## Mode of Action of *P. gingivalis* during Periodontitis

*Porphyromonas gingivalis* is an anaerobic Gram-negative bacterium involved in the onset of inflammation and tissue destruction during periodontal disease. It can be found in small numbers in the oral cavity of healthy individuals (1, 2). Pathology occurs when *P. gingivalis* binds to and accumulates on the tooth surface, leading to the development of a mixed biofilm, the expansion of the bacteria into the gingival sulcus, and the formation of a periodontal pocket (3). Inside this periodontal pocket lies the gingival crevicular fluid, an inflammatory exudate – source of essential nutrients for *P. gingivalis* growth – present in low abundance in healthy individuals, but drastically increased during gum inflammation. In this milieu, *P. gingivalis* invades gingival epithelial cells via the binding of its fimbriae to β1 integrin on the host cell surface followed by a rearrangement of the host actin cytoskeleton (4, 5). It then blocks apoptosis through the PI3K/Akt and JAK/Stat pathways, allowing intracellular bacterial proliferation (6, 7). In addition, it inhibits IL-8 expression by epithelial cells, creating what is known as the “local chemokine paralysis” (8). This mechanism induces a delay in neutrophil recruitment, which allows the proliferation of bacteria in this new niche, leading to an alteration of the subgingival microbiome with respect to its composition and total bacterial count (9, 10). The emergence of this dysbiotic assembly of microorganisms is believed to be partly responsible for the pathology observed. This is supported by findings in a murine model of *P. gingivalis*-induced periodontitis, where *P. gingivalis* was shown to contribute to periodontal bone loss by reshaping the normal commensal microbiota, while it failed to induce bone loss in germ-free animals (11). Its activity as a “keystone pathogen” may well arise directly from its atypical LPS, which does not activate TLR4 – acting either as a weak TLR4 agonist or even a TLR4 antagonist according to the local inflammatory state – and rendering it immunologically silent, potentially facilitating the initiation of the colonization (12).

## Manipulation of the Complement System by *P. gingivalis*

Early studies documented the activation and regulation of complement components in the gingival crevicular fluid where complement is believed to be present at 70% of its serum concentration (13, 14). *P. gingivalis* has developed different strategies to evade killing by the complement system. First, its surface anionic polysaccharide confers *P. gingivalis* serum resistance (15). Moreover, two types of cysteine proteases – known as gingipains – are produced by *P. gingivalis*: the lysine specific Kgp and the arginine specific RgpA and RgpB (16). While these proteases take part in the destruction of the extracellular matrix, they are also able to cleave the complement components C1, C3, C4, and C5, as well as to capture C4b-binding protein (17–20). This leads to the inhibition of complement activation, but intermittently also to a local accumulation of the anaphylatoxin C5a, the only bioactive fragment present after the actions of gingipains (20). While the massive degradation of complement proteins does not directly benefit complement resistant *P. gingivalis*, it could allow the colonization and proliferation of other bacterial strains with a higher sensitivity toward complement killing.

The local gingipain-induced accumulation of C5a at the site of infection then activates C5aR. C5aR^−/−^ mice have been shown to be resistant to age dependent as well as *P. gingivalis*-induced experimental periodontitis (11, 21). Similarly, periodontal inflammation and subsequent bone loss could nearly be abrogated by treating conventional wild-type animals with a C5aR antagonist, underlining the important role played by this anaphylatoxin during periodontitis (22). In neutrophils, *P. gingivalis* has been shown to inhibit bacterial killing in a Mal/PI3K, C5aR-, and TLR2-dependent manner (23). This could explain the increase in anaerobic oral bacteria and the change in microbiota observed after infection with *P. gingivalis* in conventional, but not C5aR^−/−^ mice (11). In macrophages, a C5aR-TLR2 crosstalk has been demonstrated to activate the cAMP-dependent PKA pathway, leading to a reduction of intracellular nitric oxide, which permits intracellular bacterial survival (19). The presence of CXCR4 activation further accentuated this synergism (19, 24). This C5aR-TLR2 crosstalk seems particularly important in understanding how *P. gingivalis* can directly dampen the immune response in an already immunologically tolerant tissue such as the mucosa. In addition, C5aR activation in macrophages inhibits the TLR2-induced IL-12p70 production (21).

The interaction of *P. gingivalis* fimbriae with TLR2 leads to the inside-out activation of the β2 integrin CR3 (CD11b/CD18) via PI3K (25). Direct interaction of *P. gingivalis* fimbriae with the chemokine receptor CXCR4 similarly results in CR3 activation (26). In macrophages, *P. gingivalis* uses this TLR2-activated CR3 as a port of entry as well as to survive intracellularly (25). In fact, inside-out activation of CR3 has been shown to suppress IL-12p70 production in macrophages (21, 25, 26). Also, the pro-inflammatory cytokines IL-1β, IL-6, and TNFα, known to induce bone resorption, are up-regulated in a C5aR/TLR2- and CR3-dependent manner by *P. gingivalis* (21, 23, 27). The resulting inflammatory breakdown products may then further strengthen the dysbiosis as recently suggested by a study underlining the inflammophilic character of the periodontitis-associated microbiota (28). Taken together, these results highlight the role played by the complement system during periodontitis: *P. gingivalis* manipulates the host complement components to escape immune clearance, colonize its new niche, and reshape the local microbiota.

## Antimicrobial Peptides of the Oral Cavity

The oral cavity is home to various peptides with antimicrobial activity, secreted by epithelial cells, neutrophils, and salivary glands. Their expression often increases during periodontitis [reviewed in Ref. (29)]. One of these molecules, the cathelicidin LL-37, plays a major role in oral health, as illustrated by the severe periodontitis observed in patients suffering from either Kostmann or Papillon–Lefèvre syndromes, two rare conditions characterized by the absence of mature bioactive forms of LL-37 (30, 31). Various studies have nevertheless suggested that cathelicidins only possess a very limited direct microbicidal activity *in vivo* and instead exert a plethora of immunomodulatory effects [reviewed in Ref. (32)]. More recently, LL-37 has been shown to promote phagocytic uptake by macrophages, which could be used at its advantage by *P. gingivalis* (33). Alpha (HNP1-3) and beta (hBD1-3) defensins are another class of antimicrobial peptides present in the gingival crevicular fluid (29). During periodontitis, the expression of cathelicidins, α, and β defensins is increased in the gingival crevicular fluid, most particularly in the presence of *P. gingivalis* (34–36). However, *P. gingivalis* has been shown to be highly resistant to killing by LL-37 *in vitro*. Similar observations were made for defensins, suggesting that the higher antimicrobial activity observed during periodontitis may have very little direct effect on *P. gingivalis*, but most probably has a major impact on other more susceptible bacteria (36–38). This could represent another way by which *P. gingivalis* shapes the local microbiota thereby selecting for periodontopathic strains, non-periodontopathic strains having been shown to be more susceptible to the activity of antimicrobial peptides (36, 38). Importantly, LL-37 can act as a pro-inflammatory trigger during periodontitis. In fact, as well as being a chemoattractant for neutrophils expressing FPRL1 receptor, LL-37 was demonstrated to induce the production of leukotriene B4 (LTB_4_), a potent chemotactic agent, in human neutrophils via binding to the cathelicidin receptor FPR2/ALX (39, 40). LTB_4_ can then trigger LL-37 release by neutrophils in an autocrine manner, thus creating a pro-inflammatory loop eventually leading to bone tissue destruction. This inflammatory response is eventually dampened by lipoxin A_4_ – a ligand for the FPR2/ALX receptor produced during the resolution phase of inflammation (39, 41, 42). Determining copy number variation in antimicrobial peptides and screening for relevant SNP may help to stratify those at risk of developing aggressive periodontitis who would benefit from early periodontal management (43–45).

## Evasion from the Oral Cavity: Link to Cardiovascular Diseases

Numerous studies have associated chronic periodontitis with various diseases, such as rheumatoid arthritis, diabetes, and cardiovascular diseases (46–48). Similarly, *P. gingivalis* has been observed at other sites than the oral cavity (49–51). While the exact mechanism used by *P. gingivalis* to reach distant anatomical locations has not yet been defined, *P. gingivalis* has been shown to survive intracellularly in macrophages, epithelial, endothelial, and smooth muscle cells and to be able to spread from one cell to another (4, 19, 25, 52). *P. gingivalis* could therefore potentially use these cells as means of transportation to travel to peripheral tissues.

Atherosclerotic disease has long been viewed as a manifestation within disease complexes such as metabolic syndrome, renal failure, and other chronic inflammatory conditions. The atherosclerotic plaque is a site of inflammation within the arterial intima, where inflammatory cells and lipids accumulate. Viable periodontic pathogens, including *P. gingivalis*, have been found in atherosclerotic plaques in mice and in humans (49–51). Antimicrobial peptides and complement activation products are both constituents of the plaques (53–55). The abilities of *P. gingivalis* to manipulate the complement and the antimicrobial systems in remote location could putatively contribute to the progression of atherosclerosis. In fact, *P. gingivalis* has been shown to accelerate plaque formation in an apolipoprotein E^−/−^ mouse model (56).

C5a is present in atherosclerotic plaques and acts as a proatherogenic molecule (57). While it does not seem to play a role in the initial development of the pathology, C5a has been shown to promote apoptosis in endothelial and smooth muscle cells as well as to induce the expression of the metalloproteases MMP1 and MMP9 in macrophages in atherosclerotic plaques. This leads to the degradation of the extracellular matrix and to the rupture of the plaque (57–59). Similarly, reduced plaque size was observed after treatment of ApoE^−/−^ mice with a C5aR antagonist (60).

Elevated expression level of LL-37 has been reported in atherosclerotic lesions, where it is thought to modulate the local immune response and induce apoptosis in vascular smooth muscle cells (54, 61). The presence of LL-37-resistant *P. gingivalis* in the lipid plaque could lead to an increase of the local concentration of antimicrobial peptides. Together with the gingipain-dependent local accumulation of C5a in the vicinity of *P. gingivalis*, this could be responsible, at least in part, for the exacerbated pathophysiology observed in the mouse model as well as in human disease.

## Potential Therapeutics

The molecular actions involving complement and antimicrobial peptides (and others) in the oral cavity are now well known but the systems are not easily amenable to therapeutic targeting. Treatments against periodontitis consist mainly on reducing the formation of bacterial plaque in the oral cavity using physical and chemical forces. Antibiotics may be given as a short course but they usually only accompany periodontal treatment, as they have difficulties to penetrate periodontal biofilms. Various isolates of oral bacteria such as *Lactobacilli* have been shown to reduce *in vitro* the growth of different periodontopathogens including *P. gingivalis* (62, 63). Clinical trials confirmed the potential of these probiotic agents to be used as a complement to periodontal treatments (63–65). Vitamin D supplementation with its beneficial effect on bone mineralization and its anti-inflammatory potential (inhibition of IL-6, IL-8, and TNFα) may as well be an additional therapy to consider (66, 67). Another option consists on the use of proresolving mediators; in fact, topical applications of the resolvin RvE1 molecule were able to reduce and even to some extent restore periodontitis-associated bone loss in a rabbit model of experimental periodontitis (68, 69). However, the most promising therapy, to date, remains the periodontal vaccines as immunization has been shown to protect against experimental periodontitis in different animal models and could potentially prevent the overt inflammation observed in associated diseases (56, 70).

## Conclusion

**Figure 1:**
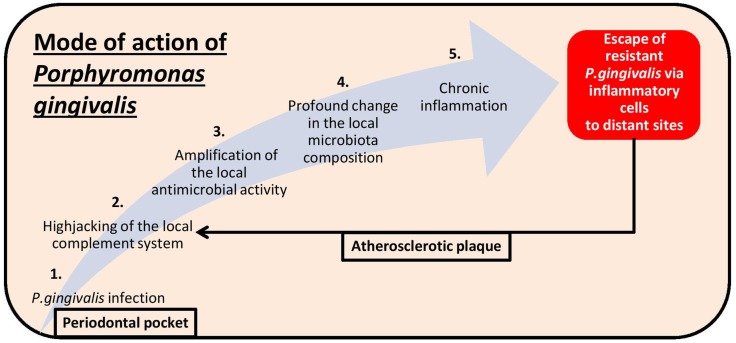
**Pathomechanistic sequence of events leading to periodontitis following *Porphyromonas gingivalis* infection (light blue arrow) as well as to the exacerbated pathophysiology observed in atherosclerosis plaques after evasion of the bacteria from the oral cavity (black arrow)**.

*Porphyromonas gingivalis* is a good example of a bacterium able to shape the composition of its microbial environment and to subvert the immune system toward chronic inflammation (Figure 1). Evidence of periodontopathogens in atherosclerotic plaques implies a direct role – which might have justified the recent broad population health advice of increasing oral hygiene – but the concomitant presence of oral and gut commensals in biopsies of atherosclerotic arteries begs as well the question of how leaky our mucosal tolerance barrier is.

## Conflict of Interest Statement

The authors declare that the research was conducted in the absence of any commercial or financial relationships that could be construed as a potential conflict of interest.
